# Cyanobacteria toxins in the Salton Sea

**DOI:** 10.1186/1746-1448-2-5

**Published:** 2006-04-19

**Authors:** Wayne W Carmichael, RenHui Li

**Affiliations:** 1 Wright State University Department of Biological Sciences 3640 Colonel Glen Highway Dayton, Ohio 45435, USA; 2 Institute of Hydrobiology Chinese Academy of Sciences Wuhan, Hubei 430072, China

## Abstract

**Background:**

The Salton Sea (SS) is the largest inland body of water in California: surface area 980 km^2^, volume 7.3 million acre-feet, 58 km long, 14–22 km wide, maximum depth 15 m. Located in the southeastern Sonoran desert of California, it is 85 m below sea level at its lowest point. It was formed between 1905 and 1907 from heavy river flows of the Colorado River. Since its formation, it has attracted both people and wildlife, including flocks of migratory birds that have made the Salton Sea a critical stopover on the Pacific flyway. Over the past 15 years wintering populations of eared grebe (*Podiceps nigricollis*) at the Salton Sea, have experienced over 200,000 mortalities. The cause of these large die-offs remains unknown. The unique environmental conditions of the Salton Sea, including salinities from brackish freshwater at river inlets to hypersaline conditions, extreme daily summer temperatures (>38°C), and high nutrient loading from rivers and agricultural drainage favor eutrophic conditions that encourage algal blooms throughout the year. A significant component of these algal blooms are the prokaryotic group – the Cyanophyta or blue-green algae (also called Cyanobacteria). Since many Cyanobacteria produce toxins (the cyanotoxins) it became important to evaluate their presence and to determine if they are a contributing factor in eared-grebe mortalities at the Salton Sea.

**Results:**

From November 1999 to April 2001, 247 water and sediment samples were received for phytoplankton identification and cyanotoxin analyses. Immunoassay (ELISA) screening of these samples found that eighty five percent of all water samples contained low but detectable levels of the potent cyclic peptide liver toxin called microcystins. Isolation and identification of cyanobacteria isolates showed that the picoplanktonic *Synechococcus *and the benthic filamentous *Oscillatoria *were dominant. Both organisms were found to produce microcystins dominated by microcystin-LR and YR. A laboratory strain of *Synechococcus *was identified by PCR as being closest to known marine forms of this genus. Analyses of affected grebe livers found microcystins at levels that may account for some of the acute mortalities.

**Conclusion:**

The production of microcystins by a marine *Synechococcus *indicates that microcystins may be a more common occurrence in marine environments – a finding not recognized before this work. Further research should be done to define the distribution of microcystin producing marine cyanobacteria and to determine exposure/response effects of microcystins and possibly other cyanotoxins in the Salton Sea. Future efforts to reduce avian mortalities and remediate the Salton Sea should evaluate vectors by which microcystins enter avian species and ways to control and mitigate toxic cyanobacteria waterblooms at the Salton Sea.

## Background

Beginning in the 1990's, massive avian and fish epornitics have occurred in the Salton Sea and over 200,000 eared grebes have died [1]. The largest single epizootic occurred in 1992 when an estimated 155,000 birds, primarily eared grebes (*Podiceps nigricollis*), died from an undiagnosed cause. The cause of these massive grebe epornitics remains unknown, although several diseases such as avian botulism and avian cholera have been diagnosed [2]. Algal biotoxins, especially those produced by dinoflagellates, have also been listed as a possible contributing cause [3], but none of the known dinoflagellate toxins has been identified to date. Preliminary results from analyses of phytoplankton samples and eared grebe tissues collected at the sea in the early 1990's identified microcystins produced by cyanobacteria [[4,5], internal reports to USGS]. Eared grebes winter on the Salton Sea, therefore the epornitic usually occurs annually during the late winter and early spring. Eared-grebe tissues collected from bird mortalities in the Salton Sea during 1992 – 94 had the cyanotoxin – microcystin – in concentrations high enough to cause acute toxicity. Enzyme Linked Immunosorbent Assay (ELISA) measured values of microcystins, in 25 samples of liver, gizzard and upper gastrointestinal tract showed levels of microcystin in liver as high as 700 ng/g. This is well above the known levels of microcystin in liver that could cause acute lethality (about 200 ng/g) [6]. Forty-nine water samples of phytoplankton, provided by the US Fish and Wildlife Service, collected in 1995–96, from the Salton Sea, contained levels of microcystins that ranged from negative to 2 ppb [Carmichael, internal reports to the USGS]. These levels are low and not likely to cause acute toxicity. However the toxin was associated with an organism smaller than 5 microns. These results suggest a small planktonic cell or picoplankton (i.e. the fresh/brackish/marine cyanobacterium *Synechococcus*) may be present in the Sea that produces microcystin. Small planktonic cyanobacteria are known to produce microcystins [7]. This background data formed the basis for the hypothesis of this project: Microcystins contribute to the eared grebe mortalities on the Salton Sea and that a significant source of organism(s) producing microcystins is to be found in the picoplankton.

The Salton Sea has salinities that vary from freshwater/brackish water at the major river outlets to hypersaline conditions in the sea proper, extreme daily summer temperatures (>40°C), and high nutrient loading (eutrophic conditions) from rivers and agricultural drainages which encourages algal blooms throughout the year [8]. These algal blooms could produce biotoxins that contribute to the massive fish and avian mortalities at the Salton Sea. In addition very little is known about algal species that survive under the existing conditions of Salton Sea. The combination of extreme summer temperatures, various saline water conditions, and nutrient loading provide a unique opportunity to investigate algal species in the Salton Sea area. Because very little is known about these algal species, and their biotoxins, there is high potential for new species and biotoxin identification that may contribute to an understanding of the high fish and avian mortalities. The purpose of this study is to collect water samples for the identification of algal species and biotoxins (cyanotoxins only), and to determine the presence of cyanotoxins in eared grebe tissues.

Bloom and mat-forming cyanobacteria in fresh, brackish and marine waters produce a wide variety of toxins including hepatotoxins, neurotoxins and dermatotoxins (Table 1). Hepatotoxins are the most frequently found cyanobacterial toxins in fresh and brackish waters worldwide. The most common group, the microcystins and nodularins, are cyclic peptides consisting of seven or five amino acids respectively. About 70 different structural variants of microcystins and a few nodularins are known. They vary in potency from highly toxic to non-toxic depending on the specific chemical structure, though most are very toxic [4,5,9].

**Table 1:** Name and producer organism for the cyanotoxins

NAME	PRODUCED BY
**Neurotoxins**	
Anatoxin-a Homo-Anatoxin-a	*Anabaena, Aphanizomenon, Oscillatoria*
Anatoxin-a(s)	*Anabaena, Oscillatoria (Planktothrix)*
Paralytic Shellfish Poisons (Saxitoxins)	*Anabaena, Aphanizomenon, Cylindrospermopsis, Lyngbya*
**Liver Toxins**	
Cylindrospermopsin	*Aphanizomenon, Cylindrospermopsis, Raphidiopsis, Umezakia*
Microcystins	*Anabaena, Aphanocapsa, Hapalosiphon, Microcystis, Nostoc, Oscillatoria, Planktothrix*
Nodularins	*Nodularia *(brackish water)
**Contact Irritant-Dermal Toxins**	
Debromoaplysiatoxin, Lyngbyatoxin	*Lyngbya *(marine)
Aplysiatoxin	*Schizothrix *(marine)
From Carmichael [28]	

Microcystins have been characterized from all of the most cosmopolitan cyanobacteria genera including *Anabaena, Microcystis, Oscillatoria, Planktothrix, Nostoc, Anabaenopsis *and *Hapalosiphon *while nodularin is found in the brackish water species *Nodularia spumigena*. An alkaloid hepatotoxin cylindrospermopsin is produced by *Cylindrospermopsis raciborskii*, *Umezakia natans, Raphidiopsis curvata *and *Aphanizomenon ovalisporum *[10].

Three groups of cyanobacterial neurotoxins are known: (i) anatoxin-a and homoanatoxin-a, which mimic the effect of acetylcholine, (ii) anatoxin-a(s), which is an anticholinesterase and (iii) saxitoxins, which block nerve cell sodium channels. Anatoxin-a has been found in *Anabaena, Oscillatoria *and *Aphanizomenon*, homoanatoxin-a in *Oscillatoria*, anatoxin-a(s) in *Anabaena*, and saxitoxins in *Aphanizomenon, Anabaena, Lyngbya *and *Cylindrospermopsis*. Sixteen confirmed saxitoxins from cyanobacterial samples have been reported, some of which (the decarbamoyl-gonyautoxins) may be chemical break-down products in some species.

In marine waters, benthic cyanobacteria such *as Lyngbya, Oscillatoria and Schizothrix *may produce toxins causing severe dermatitis among swimmers in contact with cyanobacteria. Aplysiatoxin and debromoaplysiatoxins are protein kinase C activators and potent tumor promoters. Lyngbyatoxin A exposure has caused severe oral and gastrointestinal inflammations in humans. *Trichodesmium *sp. occurring in tropical seas are known to contain an as yet uncharacterized neurotoxin. Though comparatively poorly studied, cell wall components, particularly lipopolysaccharide endotoxins (LPS), from cyanobacteria may contribute to human health problems associated with exposure to mass occurrences of cyanobacteria. Cyanobacteria are also known to produce several other bioactive compounds, some of which are of medical interest, as well as compounds toxic to other cyanobacteria, bacteria, algae and zooplankton.

The cyanotoxins are collectively responsible for continued widespread poisoning of wild and domestic animals and human fatalities. Avian mortalities from cyanotoxins have been reported since the early 1900's [11]. More recent reports of microcystin induced avian mortalities are from great blue herons [12] and flamingos [13]. While these events in themselves document the continued concern for cyanotoxins it is the emerging business of fresh and marine aquaculture organisms that could be affected most in the United States. Anthropogenic inputs from agriculture, industry, and municipal wastes coupled with heavy nutrient loading of use waters by the aquaculture industry are stimulating blooms of toxigenic cyanobacteria in fresh and marine aquaculture farms. Cyanotoxins particularly microcystins have already had significant impacts on selected aquaculture organisms including salmon, stripped bass, shrimp and catfish (Table 2). The most well-defined is the loss of Atlantic net-pen reared salmon from microcystins produced by as yet unknown organisms [14]. These losses have continued since 1991 and have caused salmon losses in the state of Washington [Carmichael unpublished data].

**Table 2:** Examples of recent environmental and health problems with toxic cyanobacteria

Toxin/Organism	Problem	Reference
Microcystins (organism unknown)	Associated with deaths of Eared Grebes-Salton Sea, Calif.	[28]
Anatoxin-a(s) (*Anabaena *sp)	Death of Crested Grebe, Black-necked Grebe, Coot, domestic ducks and geese Denmark and USA	[29] [30]
Microcystin (organism unknown)	Net-Pen liver disease of Mari-cultured Atlantic Salmon: British Columbia, Canada and Washington, USA	[14] Carmichael (unpubl. data)
Microcystin (organism unknown)	Intestinal lesions of mari-cultured penniped shrimp: Hawaii, USA and Columbia, South America	Carmichael (unpublished)
Microcystin (organism unknown)	Acute lethal liver disease of aqua-cultured stripped bass: California, USA	Carmichael (unpublished)
Microcystin (organism unknown)	Acute lethal liver disease of aqua-cultured pond-raised catfish: Mississippi USA	[31]
Microcystins (*Microcystis aeruginosa*)	Acute non-lethal toxicity in natural populations of trout and carp: England and Australia.	[32] [33]
Microcystins	At least 52 human fatalities from use of contaminated municipal water in a hemodialysis clinic: Pernambuco, Brazil	[6] [34]
Microcystins	Great Blue Heron mortalities	[12]

## Results

ELISA analyses of the waterbloom field samples showed that almost all had measurable levels of microcystin but that no sample had microcystin levels that exceeded more than 100 μg/L. This was due to the presence of salt which contributed to the dry weight levels and to the mixed biomass in which not all was toxigenic cyanobacteria. Two examples of the ELISA-microcystin values found are given in Table 3 (low and high end of the microcystin values). The PPIA assay did not respond well to the presence of salt in these samples and results were not satisfactory for reporting. It is possible that with more work the sample preparation method can be improved allowing high salt samples to be tested for microcystins by PPIA.

**Table 3:** ELISA MCYST results for shipment #1-(11/29/99) and shipment #2- (09/20/00)

Sample	Shipment#/Date	sample gdw g	ELISA Avg (n = 3) μg/gdw	STD
A1	#1-11-29-99	0.1149	0.035	0.004
B1	#1-11-29-99	0.1092	0.040	0.003
E1	#1-11-29-99	0.2727	0.024	0.002
F1	#1-11-29-99	0.1405	0.085	0.004
G1	#1-11-29-99	0.1355	0.020	0.002
H1	#1-11-29-99	0.1625	0.030	0.003
E2	#1-11-29-99	0.165	0.056	0.008
F2	#1-11-29-99	0.3335	0.047	0.006
G2	#1-11-29-99	0.1391	0.027	0.001
H2	#1-11-29-99	0.1139	0.056	0.000
B2-4	#1-11-29-99	0.1879	0.033	0.002
A2-4	#1-11-29-99	0.1221	0.056	0.005
B1	#1-11-29-99	0.3017	0.052	0.004
A1 (Grab)*	#2-09-20-00	0.211	98.5	0.04
A1 (Tow)	#2-09-20-00	0.223	68.4	0.03
A2 (Grab)	#2-09-20-00	0.235	94.2	0.01
A2 (Tow)	#2-09-20-00	0.168	85.7	0.03
O1 (Grab)	#2-09-20-00	0.196	77.6	0.04
O2 (Tow)	#2-09-20-00	0.132	54.5	0.08
W1 (Grab)	#2-09-20-00	0.198	99.2	0.04
W2 (Tow)	#2-09-20-00	0.223	43.1	0.09
N1 (Grab)	#2-09-20-00	0.178	23.4	0.02
N1 (Tow)	#2-09-20-00	0.230	17.4	0.03
N2 (Grab)	#2-09-20-00	0.320	67.8	0.01
N2 (Tow)	#2-09-20-00	0.334	87.3	0.02

* A=Alamo River; O = Open water; W = White Water River; N = New River

Red Hill Marina (A1), Lack Road (B1), New River (E1), Midlake b/w New & Alamo (F1), Alamo River (G1), Pelican Feeding Site (H1), New River Sediment (C1), New River Sediment (D1), Red Hill Marina (A2-4), Lack Road (B2-4), New River (E2), Midlake b/w New & Alamo (F2), Alamo River (G2), Pelican Feeding Site (H2)

A1, B1, E1, F1, G1, H1 = plankton tow; C1, D1 = sediment sample; rest of samples are all surface grabs gdw = gram dry weight

Not all phytoplankton were identified in the water samples. Emphasis was given to the toxigenic cyanobacteria. A summary of the main phytoplankton found in the water samples is given in Table 4. These cyanobacteria are similar to those reported in other work on phytoplankton in the Salton Sea. Wood et al. [15] also listed *Oscillatoria *and *Synechococcus *as being very common in their samples which were obtained during January and June of 1999. Their work and that of others looking at Salton Sea phytoplankton did not investigate for the presence of cyanotoxins such as the microcystins.

**Table 4:** Dominant phytoplankton in the Salton Sea. Samples for the period november 1999 to April 2001

**Cyanobacteria**
Geitlerinema
Lyngbya
Merismopedia
Oscillatoria
"Picoplankton" Synechococcus
**Chlorophyta**
Crucigenia
**Diatoms**
Cyclotella
Cylindrotheca
Navicula
Pleurosigma
**Dinoflagellates**
Gymnodinium
Gyrodinium

Selected phytoplankton samples from almost all shipments were plated onto agar plus medium and cultured for isolation of cyanobacteria that may be cyanotoxin producers. During the course of this project 100 samples were plated and approximately 150 unialgal cultures made. The isolates were dominated by the filamentous *Oscillatoria *and the picoplanktonic *Synechococcus*. Because the duration of the study was relatively short cultures tested were those that responded quickly to the particular culture conditions in our facilities. The culture types found in our study were again similar to those obtained in a study by Wood et al. [15]. Of these cultures 50 of the better growing isolates were processed and tested by ELISA for microcystin. Thirty four of the 50 cultured isolates were positive for microcystins. Values were low and ranged from detectable (0.147 μg/L) to 1 μg/L. Four of these 34 cultures were identified as *Synechococcus *and all four were positive for microcystins by ELISA and LC/MS. Only strain SS-1, of these four strains, was identified as *Synechococcus *by PCR.

**Figure 1:**
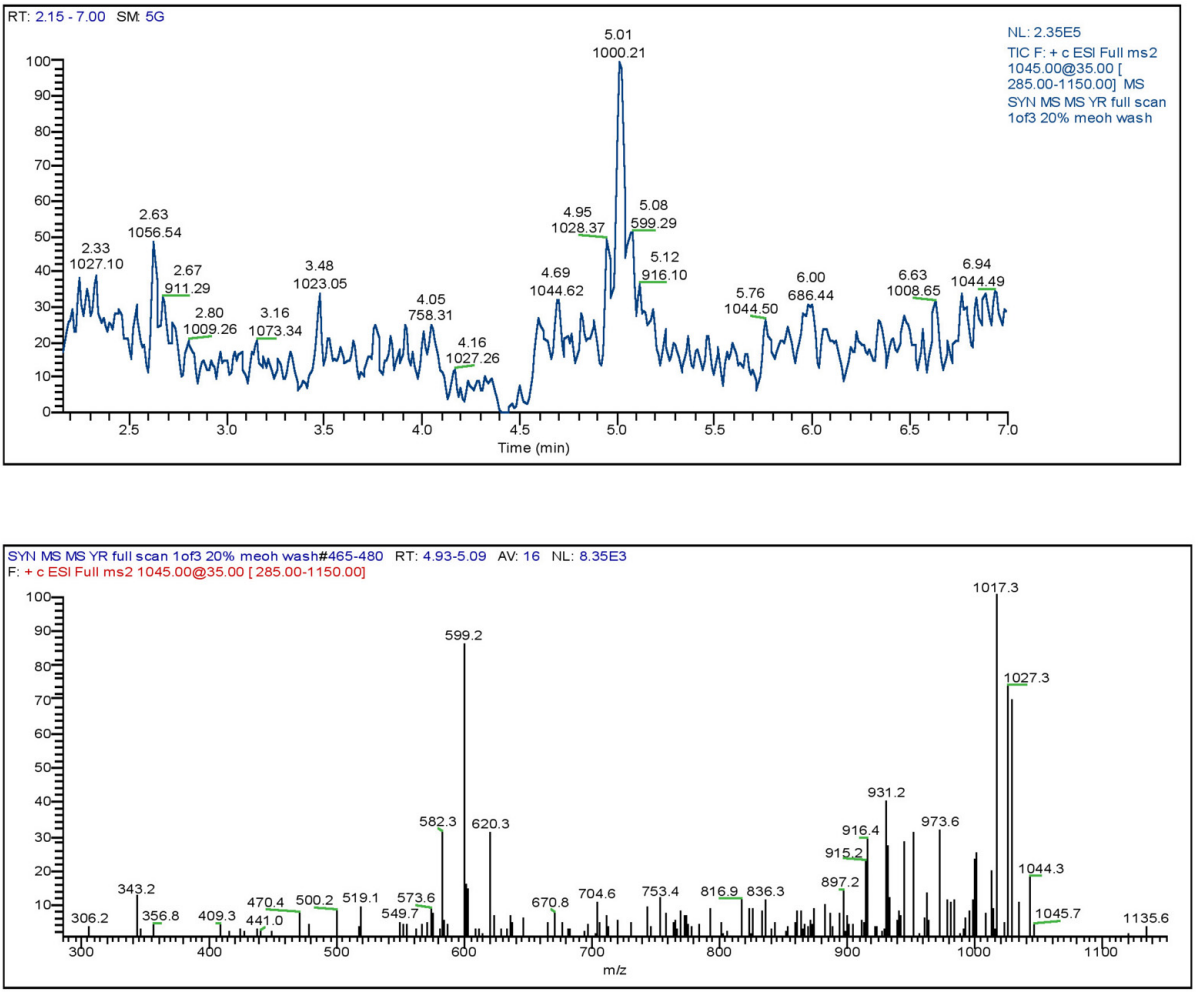
LC/MS-MS full scan of *Synechococcus *sp. (SS-1; JP-Syn)-MCYST-YR (M+H = 1045)

**Figure 2:**
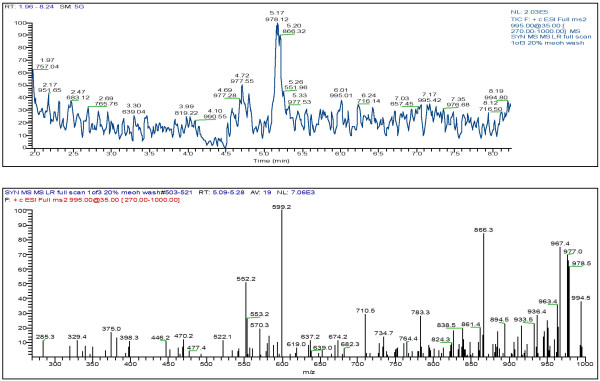
LC/MS-MS full scan of *Synechococcus *sp. (SS-1; JP-Syn)-MCYST-LR (M+H = 995)

**Figure 3:**
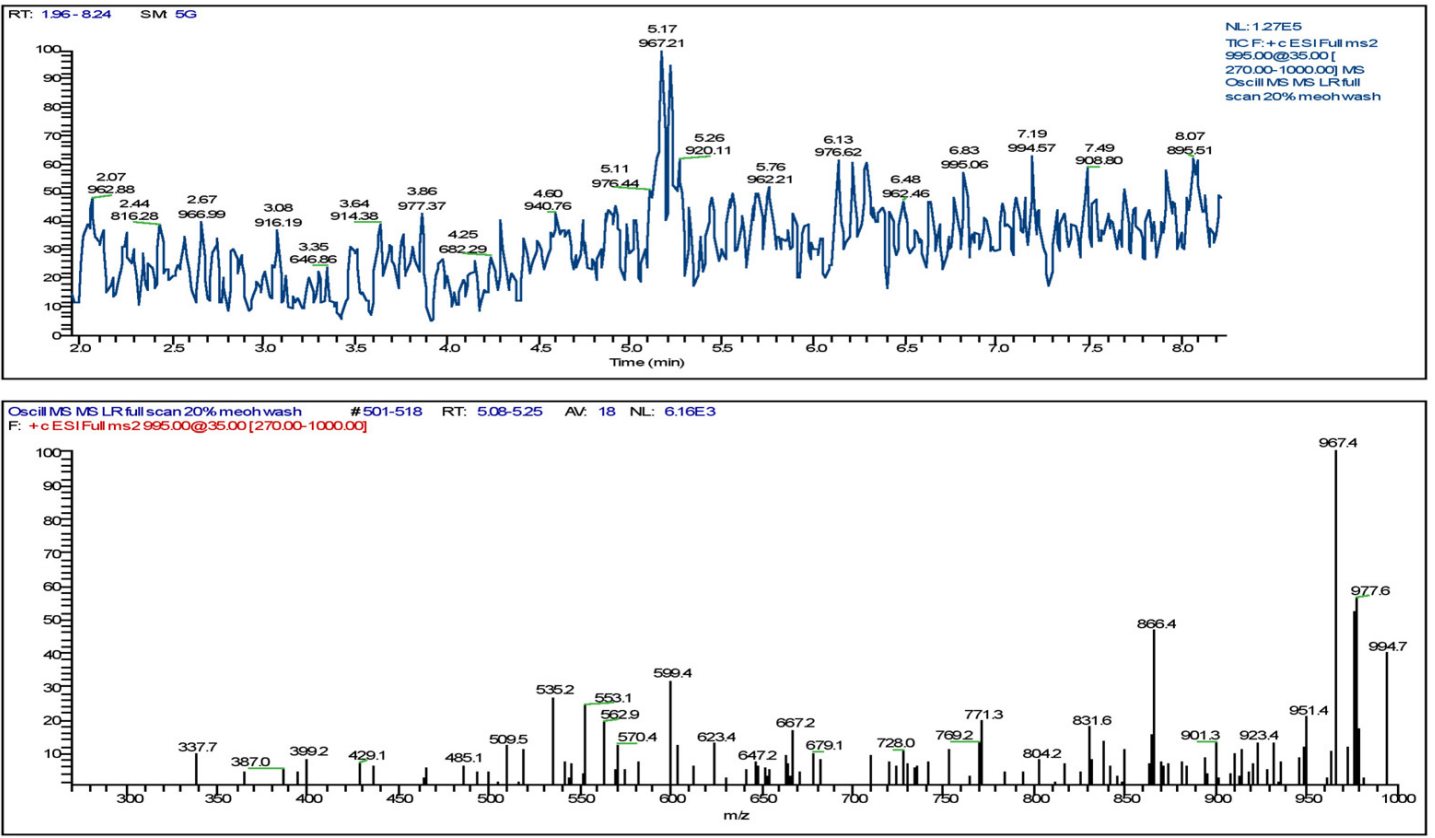
LC/MS-MS full scan of *Oscillatoria *sp. (O-1; Bloom 405)-MCYST-LR (M+H = 995)

LC/MS analyses of selected cultures was also used to confirm the presence of microcystin production. Since the picoplankton have potential to be a significant source of microcystin year round in the Salton Sea the presence and type of microcystin in these cultures was analyzed. Figure 1 and 2 shows that microcystin-YR and LR, respectfully, were present in strain SS-1 of *Synechococcus *while strain O-1 of *Oscillatoria *contained microcystin-LR (Fig. 3).

**Figure 4:**
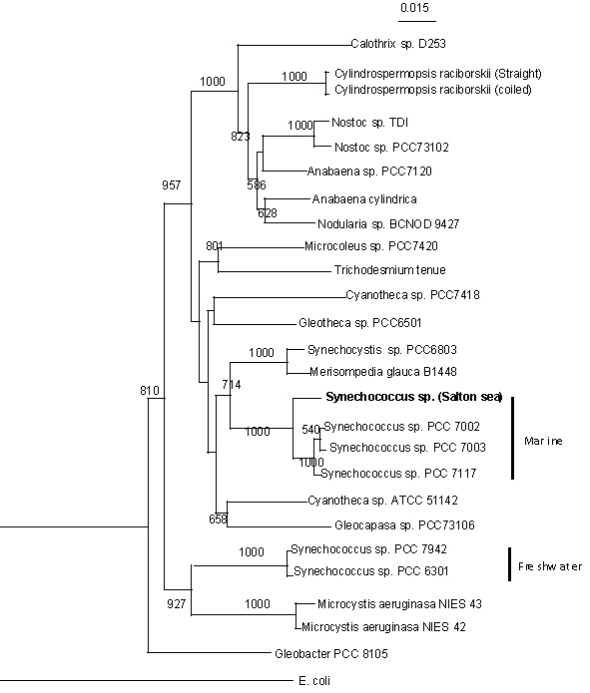
Dendrogram for *Synechococcus *(SS-1; JP-SYN). Local bootstrap probabilities are given at the nodes

Cyanobacterial 16S rRNA gene sequences available from GenBank and those examined in the study were aligned using the multiple sequence alignment tools in CLUSTAL W version 1.7 [16]. This was followed by conversion to a distance matrix. The distance matrix was used to reconstruct a phylogenetic tree (Fig. 4) by the neighbor-joining (NJ) algorithm of CLUSTAL W version 1.7, with multiple substitutions corrected and positions with gaps excluded. The seed number for random number generation and the number of bootstrap trials were set to 111 and 1000, respectively.

*Access number*. Partial 16S rRNA gene sequences of the strains used in this study can be obtained in Genbank using access number: DQ455751. Results of the sample culturing, microcystin analyses and generation of the dendrogram show that microcystin producing *Synechococcus *was a member of the Salton Sea phytoplankton during the course of this study. From the dendrogram results and comparison with other GenBank gene sequences we found that the Salton Sea *Synechococcus *strain tested is closer to marine strains of *Synechococcus *than to freshwater strains of this genus. This is significant since it shows that marine cyanobacteria can produce Microcystins, a finding not demonstrated until this study. This has implications for the possible presence of microcystins in other marine environments and may make microcystin a marine HAB as well as a freshwater HAB toxin.

Five of the 20 shipments contained eared grebe tissues-liver, intestine and stomach contents. Table 5 gives the values for microcystin in these tissues as measured by ELISA. Compared with other cases of microcystin poisoning the amounts found in these eared grebe livers are in the range for acute toxicity and possibly acute-lethal toxicity. For example the average microcystin value in 52 liver samples from 39 human victims who died from microcystin exposure during dialysis was 223 ng/g [6]. In addition intraperitoneal dosing of mice showed that 125 ng/g of microcystin-LR was detectable by ELISA in the livers of mice exposed to a lethal intraperitoneal injection (100 μg/kg).

**Table 5:** Salton Sea – Grebe samples

		MCYST	MCYST
Sample Site	Description	ELISA ng/g dry weight tissueliver	ELISA intestineng/g
27 grebe tissue samples	Case#4586 #005-13	005(BDL);006(TR);007(10) 008(BDL); 009(BDL);010(5) 011(BDL);012(10);013(BDL)	005(BDL);010(BDL) 013(BDL)
15 grebe tissues	Case#4586 #014-18	014(20);015(36);016(27) 017(44);018(32)	014(BDL)'018(BDL)
6 grebe tissue Samples	Case #4586 #019-20	019(56);020(76)	019(10);020(20)
1 grebe tissue #23; algae sample	stomach content**		
12 grebe tissue Samples	Case#4586#021-2-4-7	021L(87);022(85);024(86) 027(57)	021(23);027(BDL)
10 Grebe tissue samples-Mono Lake – control	Case#4607#022-28	All Tissues BDL	All Tissues BDL
21 grebe tissue samples	Case #4617-001-7 intestine, liverand stomach content	001(60);002(90);003(105)004(110);005(89);006(74) 007(69)	001(20);002(40) 003(25), 007(15)

BD = below level of detection; TR = trace

**Stomach content not done due to low amount or poor matrix extraction

S# = shipment #

Microcystin types in selected liver samples were analyzed by LC/MS. Mass peaks indicative for microcystin-LR and YR were identified although the sensitivity was not low enough to confirm their presence by LC/MS-MS.

## Discussion

The cyanotoxin group microcystins should be considered as a possible contributing cause of grebe morbidities and mortalities in the Salton Sea and efforts to remediate the Salton Sea water quality should take this into account. Our study did not include controlled exposure studies to grebes and this is an obvious need for future research in order to determine the role of cyanotoxins in these toxicities. We also did not investigate the possible routes by which microcystins vector into grebe livers and intestines-another important study that should be undertaken. Finally the finding that all cultured strains of the picoplankton *Synechococcus *produces microcystin is important in itself but that in addition the PCR based genetics places it within the marine *Synechococcus *cluster is even more significant. Clearly more work is needed to extend this finding and clarify if marine *Synechococcus *producing microcystin are widespread in the Salton Sea and in other marine systems. Salinity levels may be a significant factor in selecting for the microcystin producers. In a study on the Swan River Australia, Orr et al. [17] demonstrated that laboratory cultures of microcystin producing *Microcystis aeruginosa *were more tolerant to high salt concentrations and were preferentially selected for, as salt levels were increased in the culture media.

Efforts to reduce salinity and improve water quality in the Salton Sea should consider that the genera of cyanobacteria currently in the sea are not high producers of microcystins. If salinities are lowered without also managing levels of key nutrients such as nitrogen and phosphorus other genera of cyanobacteria that produce higher levels (acute lethal) of microcystins may be selected for. These genera could include *Microcystis *and *Anabaena*. Waterblooms of these genera are typically more intense and would present a higher risk from cyanotoxin (microcystins, anatoxins, cylindrospermopsin and saxitoxins) exposures.

## Conclusion

An investigation to determine the role of cyanotoxins in grebe mortalities and morbidities on the Salton Sea was started in November 1999 and ended in April 2001. Water sampling dates varied but samples were received from every month except May, June and July of 2000. Twenty shipments were received over this 18-month period. Fifteen of the shipments contained water and phytoplankton samples while six contained grebe tissue samples (one shipment contained both water and tissue samples). Water samples containing cyanobacteria at numbers giving a visible color to the water samples (approx 10^5 ^to 10^7 ^cells/ml) were lyophilized and analyzed for microcystins by ELISA. Approximately 85% of 247 samples were positive for microcystins. Concentrations of microcystins were typically less than 100 μg/gdw. This concentration is generally less than the needed to cause acute lethal toxicity from ingestion of bloom samples by mammals.

Throughout the sampling period the majority of water samples were dominated by the filamentous genus *Oscillatoria *and the picoplanktic genus *Synechococcus*. Isolation, culture and ELISA testing for microcystin of 50 strain isolates found that microcystins were produced by all strains – although at low levels (<1 μg/gdw). The genera producing measurable levels of microcystin included mainly *Synechococcus *and *Oscillatoria*. Other positive microcystin genera included *Gloeocapsa, Phormidium, Aphanothece *and *Lyngbya*.

PCR 16S rRNA analyses was done on one strain of *Synechococcus, SS-1*. Results confirmed that this strain was *Synechococcus *and comparison with a marine cluster of *Synechococcus *supports a conclusion that this *Synechococcus *strain is more similar to marine than to freshwater members of this genus. All four *Synechococcus *isolates were shown by ELISA and LC/MS to produce the microcystins-MCYST-LR and MCYST-YR.

Microcystins were detected by ELISA in the majority of liver and intestine samples from grebes collected due to morbidity or mortality. The concentrations of microcystins were not always high enough to account for acute lethal toxicity (< 50 ng/g) but a significant number of liver samples did contain microcystin levels high enough to account for acute lethal toxicities (>50 ng/g).

Although the concentration of microcystins in the water samples and in strain isolates of cyanobacteria were not high enough to account for acute lethal toxicity, levels in grebe livers often did suggest acute or acute lethal toxicity could occur. This suggests that microcystins accumulated, by an as yet unknown vector(s), in grebe tissues to levels that could be lethal.

## Methods

### Sample collection, shipment and handling

Water samples were collected by personnel from the Salton Sea Wildlife Refuge, as defined by the needs of the project, the work schedule for the Salton Sea Wildlife Refuge personnel and at times weather and other logistical conditions. Collections started in November 1999 and ended in April 2001. Sampling dates varied but samples were received from every month except May, June and July 2000. Twenty shipments were received over this 18-month period. Fifteen of the shipments contained water and plankton samples while six contained grebe tissue samples (one shipment contained both water and tissue samples). Grebe necropsies and tissue shipments were done by SS Refuge personnel and personnel at the National Wildlife Health Center-USGS in Madison, Wisconsin (Chris Franson) after collection by Salton Sea personnel. Control grebe tissue samples were arranged by Chris Franson and were collected at Mono Lake California during October 2000.

Sample Kits used to collect and ship the toxigenic algae samples, contained:

1. (1) shipping cooler

2. large liner bag (for lining inside of cooler) and cable-tie for securing it closed

3. 500 ml-Nalgene sample bottles

4. zip-lock bag (for enclosing sample report form)

5. ice-pack

### Sample collection locations

**Figure 5:**
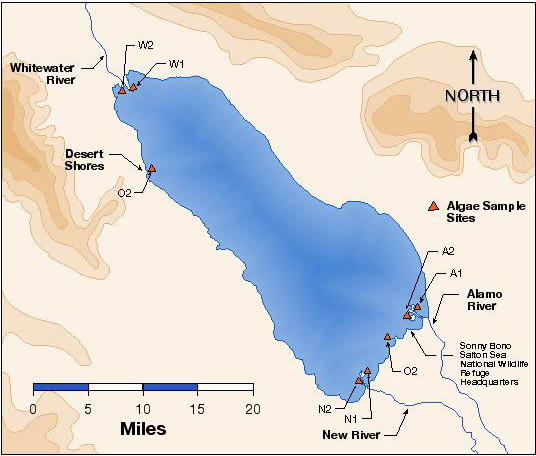
Salton Sea map showing areas of sample collections. Collections focused on inlets for rivers. A = Alamo River, N = New River, O = Open water, W = Whitewater River.

During the time that shipments 1–3 were made a transect system was used to collect samples. These transects were set by Salton Sea personnel and covered river inlets, open waters and near shore areas. Later it was determined that most phytoplankton was to be found near areas of river inlets where salinity was lowest. This was also the areas where bird mortalities were typically highest. This resulted in 4 sample sites: A=Alamo River, N=New River, O=open water and 1 location in the north, W=Whitewater River. Figure 5 gives the overall view of sample sites on the Salton Sea for this project.

### Sample receipt and processing

1. Samples were logged in and then stored at 4°C for phytoplankton analyses and at -80°C in preparation for lyophilization, extraction and toxin analyses. A typical treatment regime for these samples was as follows: 1) Samples were logged in and sample location and conditions were noted. 2) Samples were split, with most of the sample being lyophilized and a small amount kept back for phytoplankton examination and identification of major cyanobacteria present. 3) Since most of the dry weight of a sample was salt, only visible green or green brown samples were used for microcystin analyses. 4) All samples that had identifiable cyanobacteria in them, by microscopic observation, were placed into either tubes with liquid culture (CT medium plus salt) or onto agar plates containing CT medium plus salts. Other cyanobacteria growth media were tested initially including BG-11, ASM-1 and Z-8 but the CT media plus salt was found to give the best overall growth results.

### Taxonomy of Salton Sea cyanobacteria isolates

Cultured isolates were examined on a Nikon Optiphot phase microscope with phase and fluorescence optics. Taxonomy, based on morphological characters, was determined from reference to Komarek and Anagnostidis [18], Komarek and Anagnostidis [19], Desikachary [20] and Carpenter [21].

#### Culture of laboratory isolates

Isolates were made by streaking water or sediment sample on plates composed of CT+ medium and agar. Colonies were visually grouped by observation with a dissecting scope and 2–5 representatives of each type of colony were lifted from a given plate via micropipette and transferred to liquid CT+ medium in a 10 ml test tube. From the test tube cultures, representatives of each type were transferred to a 4L flask of CT+, aerated and maintained under 24 hour illumination. Cultures were harvested before senescence, spun down in a Sorvall centrifuge at 5000 rpm, the medium was poured off and the pellet was rinsed with dionized water to help remove salt. The pellet was transferred to a stainless steel pan, frozen and subsequently freeze-dried. CT medium content is as given by Watanabe and Ichimura [22]. The + refers to the addition of NaCl to the medium (7 g/L).

### Detection of microcystins

The most common of the cyanotoxins, likely to be found in this study, are the cyclic peptide hepatotoxic microcystins and nodularins. Rapid and sensitive methods now exist for detecting and monitoring these toxins in environmental samples (water, cells, sediment and animal tissue). This includes a sensitive polyclonal antibody immunoassay, developed by FS Chu at the Univ. of Wisconsin, and adapted by An and Carmichael [23] and Carmichael and An [24]. This enzyme-linked immunosorbant assay (ELISA) is sensitive to ppb and the antibody cross reacts with most of the known microcystins. This chemical assay is complimented by a colorimetric enzyme activity assay [23] that measures the inhibition of microcystin against protein phosphatase 1 and 2A. Inhibition of PP1 and 2A is the specific mechanism of action for microcystins and is directly related to microcystins toxicity. These two assays can be used to monitor and quantitate microcystins in all the various studies outlined in this proposal.

ELISA assay for the cyclic peptide microcystins (MCYST) and nodularin (NODLN). The method is based upon the polyclonal antibody method described by Chu et al. 1989, 1990 and as adapted by An and Carmichael [23]and Carmichael and An [24]. The level of sensitivity for microcystin/nodularin using this method is about 0.5 ng/ml. Values below or near this level are not considered significant. Fifty microliters (50 μl) of sample containing 500 μg of algae is used for the assay. Serial dilutions of 10^-1^-10^-4 ^(in duplicate) are used to run the assay.

Samples are run on a PP1 or 2A inhibition assay. The cyclic peptide liver toxins, microcystin and nodularin, have been shown to be specific and potent inhibitors of protein phosphatases 1 and 2A (PP1 and PP2A). Inhibition of these enzymes has been shown to be correlated with the ability of these toxins to be tumor promoters, especially liver tumor promotion. The assay is therefore useful in combination with the ELISA assay (which tests for presence of the compounds – not all of which are bioactive) as an activity assay (to measure actual toxic effect). The assay is about 1000 times more sensitive than the HPLC or mouse bioassay. Assay of PP activity was done by measuring the rate of color formation from the liberation of P-nitrophenol from P-nitrophenol phosphate using a Molecular Devices Corp., Vmax kinetic microplate reader, Palo Alto, CA. [25].

### Preparation of samples for ELISA and LC/MS

#### Microcystin analysis

Freeze dried cells were extracted in methanol at a ratio of 1 g dry weight cells to 50 mL methanol. Extracts were sonicated for 30 s and placed on a rotating table overnight. After filtration through a glass fiber filter (1.6 μm pore size), the supernatants were evaporated to dryness in a Speedvac. Extracts were resuspended in 5 mL of reagent grade water and passed through a solid phase extraction column (Isolute, IST, Glamorgan, UK) containing 500 mg C18(EC). The column was washed with 5 mL of 20% (v/v) methanol and microcystins were eluted with 10 mL of 80% (v/v) methanol. The later fraction was evaporated to dryness in a Speedvac, resuspended in 1 mL of 10% (v/v) methanol, and subjected to ELISA and LC/MS analysis.

### Extraction of tissues

Liver or intestine samples (0.5–1.0 g) were homogenized in 10 mL of hexane (Power Gen 125 tissue homogenizer, Fisher Scientific Pennsylvania, USA) at 15000 rpm using a 7 mm saw tooth generator probe (Fisher Scientific, Pennsylvania, USA). After homogenization approximately 1 ml pf ethanol was added to the sample to break up emulsion formation. The sample was slowly shaken for about 0.5 hr on a orbital shaker. After this time the hexane layer was removed, dried with a stream of air or nitrogen and reconstituted in 0.5 ml of 35% MeOH. Preparation of this sample for separation of the microcystin-containing fraction was by Isolute C18 packing/3 ml reservoir cartridge. The 100% MeOH fraction from this cartridge was dried under a stream of air and reconstituted in 1 ml of 5% MeOH. This fraction was used in the ELISA and LC/MS analyses [26].

### LC/ESI-MS conditions

Column: MetaChem Monochrom C18, 2 × 50 mm, 5 micron particle size

Mobile Phase: A) 0.1% formic acid in water B) 0.1% formic acid in acetonitrile

Gradient: 25 % B to 50% B in 5 minutes, with first minute diverted to waste

(Purge 20 column volumes with 50% B at end of run; equilibrate with 20 column volumes prior to run)

Temperature: 35 deg C (column heater to stabilize temperature)

Flow: 0.25 mL/min

Injection Volume: 20 μl

Selected Reaction Monitoring (MS/MS) Scan Experiments (minimum 3 replicates)

Limit of Detection: 300–500 pg (on column)

Limit of Quantification: 0.5–1.0 ng (on column); high ppm (ng/g -ug/g) for samples; precision ≤ 15% (trace analysis for tissue samples); ≤ 5% for algae samples

Extraction Efficiency (SPE sample prep): 90%

Ionization Suppression from Tissue Matrix: 35–40% suppression in signal response

Combined reduction in recovery/response: 50%

Expected Weight of Tissue Samples: 1–2 gr

Baseline resolution of analytes, RT repeatability +/- 0.5%

### Solid phase extraction/sample preparation

Function:

1) removal of interferences and column killers

2) desalting

3) concentration or trace enrichment of analyte

Capacity of SPE cartridge: 10–20 mg analyte+interferences/g sorbent

SPE Cartridge Volume: 1 μl solvent/1 mg sorbent

### PCR of cyanobacteria isolates

#### DNA extraction

Ten mg of lyophilized or fresh cells (harvested at exponential phase and washed three times with distilled water) were mixed with microbeads in a 2 mL screwtop polypropylene vial (1:1 with volume), and broken with a Mini-Beadbeater (Biospec Products, USA) at 5000 rpm for 1 min, The solution was then suspended in 0.5 ml of a lysing solution containing archromopeptidase 0.5 mg + lysozyme 0.75 mg/mL of 10 mM Tris-HCl buffer, pH 8.0. The samples were incubated at 37°C for 30 min. Fifty μl of 10% Tris-SDS solution (SDS in 1 M Tris, w/v) was added and the solution was well mixed. Samples were then incubated at 60°C for 5 min. Lysates were then extracted twice with 0.2 mL of water-saturated phenol and 0.2 mL of chloroform. After centrifugation (15,000 rpm, 10 min) the upper layer was removed and 50 μL of RNase solution (RNase 1 mg + RNase T1 400 units)/mL of 50 mM Tris-HCl, pH 7.5 was added. This was kept at 37°C for 20 min. This was followed by a treatment with 50 μL of proteinase K solution (Proteinase K (Sigma), 4 mg/mL in 50 mM Tris-HCl, pH 7.5) at 37°C for 10 min. The samples were treated again with 0.2 mL of phenol solution plus 0.2 mL of chloroform, and centrifuged at 15, 000 rpm for 10 min. DNA was precipitated with 0.1 volume of 3 M NaOAC and 2.5 volumes of cold 100% ethanol. Precipitated DNA was pelleted by centrifugation for 15 min at 15,000 rpm, washed with 70% ethanol, 100% ethanol, dried and stored at -20°C.

### Primer designation and polymerase chain reaction amplification

Based on cyanobacterial 16S rRNA gene sequences the two primers for amplification and sequence were; F1 (5' TAACACATGCAAGTCGAA3'), and newly designed R4N(5' CCTACCTTAGGCATCCCC 3'). The latter has a sequence showing high specificity to the family *Nostocaceae*, which was checked using a BLAST database search [26]. Polymerase chain reaction (PCR) amplification was done in a 80 μl reaction mixture using 10–20 ng genomic DNA, 0.05 units/μl Ampli *Taq *DNA polymerase, 10 × buffer containing 1.5 mM MgCl_2_, 0.2 mM dNTPs, and 0.05 μM of primers. The reaction was run in a Techne Thermal Cycler (Progene, UK) with one cycle of 94°C for 5 min.; 30 cycles of 94°C for 30s, 50°C for 30s, 70°C for 1 min, and finally 72°C for 3 min.

### Sequence analysis

PCR products were purified by applying the QIA quick DNA Remove Kit (QIAGEN, USA). This was used as the template in sequencing reactions using an Applied Biosystems; PRISM Dye Terminator Cycle Sequencing Ready Reaction Kit supplied with Ampli *Taq *DNA polymerase. The primers used for the sequencing reaction were the same as for amplification. Products of sequencing reactions were analyzed on an Applied Biosystem 310 DNA sequencer.

### Alignment and phylogenetic analyses

Cyanobacterial 16S rRNA gene sequences available from GenBank and those found in the present study were aligned using CLUSTAL W version 1.6 [27]. This was followed by conversion to a distance matrix. The distance matrix was converted to a phylogenetic tree using the neighbor-joining (NJ) algorithm of CLUSTAL W version 1.6, with multiple substitutions corrected and positions with gaps excluded. The seed number for random number generation and number of bootstrap trials were set to 111 and 1000, respectively.

## Abbreviations

ELISA enzyme linked immunosorbent assay

LC liquid chromatography

MCYST microcystin

MS mass spectrometry

BDL below detection limits

## Competing interests

The author(s) declare that they have no competing interests.
